# Dietary Intake of Patients with Parkinson’s Disease in Lithuania

**DOI:** 10.3390/nu18081302

**Published:** 2026-04-20

**Authors:** Jevgenija Guk, Rūta Kaladytė Lokominienė, Anatolij Nečiporenko, Roma Bartkevičiūtė, Albertas Barzda, Dalius Jatužis

**Affiliations:** 1 Clinic of Neurology and Neurosurgery, Institute of Clinical Medicine, Faculty of Medicine, Vilnius University, LT-03101 Vilnius, Lithuania; ruta.lokominiene@mf.vu.lt (R.K.L.); anatolij.neciporenko@gmail.com (A.N.); dalius.jatuzis@mf.vu.lt (D.J.); 2 Department of Public Health, Institute of Health Science, Faculty of Medicine, Vilnius University, LT-03101 Vilnius, Lithuania; roma.bartkeviciute@hi.lt; 3 Public Health Innovation Center, Institute of Hygiene, LT-08107 Vilnius, Lithuania; albertas.barzda@hi.lt

**Keywords:** Parkinson’s disease, micronutrients, macronutrients, dietary intake, energy intake

## Abstract

**Background**: Risk of malnutrition among patients with Parkinson’s disease (PD) can reach up to 60%, with prevalence rates as high as 24%. Dietary management for PD patients is a promising adjuvant therapy that may improve some motor and non-motor symptoms. However, there is limited information regarding nutrient intake and adherence to recommended dietary requirements in this population in Lithuania. **Methods**: In this case–control study conducted at Vilnius University Hospital Santaros Klinikos (2023–2025), usual dietary intake was assessed using two non-consecutive 24 h recalls. Intake was compared with Lithuanian Recommended Daily Intake (RDI) values. Associations between nutrient intake and selected non-motor symptoms were analyzed. **Results**: Fifty-nine patients with PD and 54 controls were included and compared with RDI; patients with PD had lower intakes of dietary fiber (20.63 g/day), vitamin A (627.15 μgRE/day), and vitamin D (3.18 μg/day), alongside a higher energy contribution from total sugars (11.31 E%). Less than half met RDI for protein, fat, polyunsaturated fatty acids (PUFA), and monounsaturated fatty acids (MUFA) energy contribution, vitamins E and C, calcium, and zinc. Compared with controls, PD patients consumed more dietary fiber, plant protein, and total carbohydrate and had a higher carbohydrate-derived energy contribution. PD patients with depressive mood consumed fewer carbohydrate, dietary fiber, plant protein, and some vitamins and minerals compared to those without. **Conclusions**: Patients with PD had higher total sugar-derived energy consumption and lower dietary fiber and vitamin D intake than the RDI. There were differences in dietary intake among PD patients with and without specific non-motor functions.

## 1. Introduction

Parkinson’s disease (PD) is the second most common chronic neurodegenerative disorder, presenting with both motor and non-motor symptoms [1]. It is slowly progressing and can last for decades, gradually increasing the disability of the affected individual and the burden on caregivers, resulting in a significant social and economic strain [2]. According to global burden of disease data, the global age-standardized incidence rate of PD was 15.63 per 100,000 population, while the age-standardized incidence rate in Lithuania was approximately 10.6 cases per 100,000 population in 2021 [3]. The Global Burden of Disease Study estimates that the number of PD cases will increase from 2021 to 2050, rising from 12 million to 25 million, due to population aging and growth [4].

Suboptimal diet has a substantial impact on non-communicable disease mortality and morbidity, and nutritional risk factors are among the main contributors to disability adjusted life years worldwide [5]. According to recent studies, the dietary intake of residents of Lithuania and other countries does not fully comply with the recommended daily intake [6,7]. However, evidence shows that a healthy diet promotes the prevention of common non-communicable diseases, such as cardiovascular disease, type 2 diabetes, and some cancers [8]. Observational studies suggest that dietary patterns like the Mediterranean diet, Mediterranean-DASH Intervention for Neurodegenerative Delay (MIND), and the ketogenic diet can positively affect both the motor and non-motor symptoms of PD [9].

It has been estimated that malnutrition prevalence among people with PD ranges from 0% to 24%, while the risk of malnutrition varies from 3% to 60% [10]. Although both weight loss and weight gain have been reported in PD patients, a recent systematic review found that PD patients have a significantly lower body mass index (BMI) than controls [11,12]. Also, sarcopenia is highly prevalent among PD patients. The range of probable sarcopenia ranges from 23.9 to 66.7%, and the prevalence of confirmed sarcopenia ranges from 2 to 31.4% [13]. Both motor and non-motor symptoms can lead to malnutrition and BMI changes in individuals with PD. In the early stages of disease, premotor symptoms such as olfactory and taste disorders, depression, cognitive impairment, and gastrointestinal symptoms may contribute to decreased nutrient intake and weight loss [14,15]. As the disease progresses, worsening motor symptoms (such as tremors, rigidity, and dyskinesias) may contribute to increased energy expenditure and subsequent weight loss [12]. Medications used to treat PD can also impact patients’ nutritional habits; for example, dopamine agonists may lead to compulsive eating [16]. Patients with PD are reported to consume high amounts of sugars and carbohydrates, which may affect non-motor symptoms, increase the need for Levodopa dosage, and impact their quality of life [17,18]. Otherwise, a protein redistribution diet, which is usually recommended for PD patients to avoid Levodopa competition with large neutral amino acids and improve its absorption in the small intestine and at the blood–brain barrier, can contribute to diminished protein intake and malnutrition [19]. Some studies suggest that diet quality can deteriorate with the PD duration and progression [17]. Weight loss due to malnutrition in PD is associated with the risk of falls, fractures, infections, and a poorer quality of life [10,12].

Some studies suggest a protective effect of various macro- and micronutrients in PD, but further investigations are warranted [20,21]. On the other hand, PD patients are at increased risk of vitamin and mineral deficiencies compared to healthy controls, which can lead to worsening health issues [22,23,24]. Otherwise, a nutrition intervention for patients with PD at risk of or experiencing malnutrition, can significantly improve quality of life [25]. Several countries have proposed guidelines for dietitians emphasizing the significance of nutritional management of PD [26,27].

Despite the significance of nutritional management in PD, information about dietary habits and nutritional intake is limited. Additionally, dietary habits may vary significantly across ethnicities [28]. Understanding dietary intake in the Parkinson’s population provides valuable insights into nutritional adherence to recommended intake levels. This information can help evaluate the necessity for dietary interventions and assist in developing specific guidelines.

Due to limited information on the nutritional intake of PD patients in Lithuania, we aimed to characterize the nutritional intake of the Lithuanian PD cohort and investigate potential associations between diet and clinical disease features.

## 2. Materials and Methods

### 2.1. Participants

This case–control observational study was conducted in the Parkinson’s disease center of Vilnius University Hospital Santaros Klinikos from January 2023 to January 2025.

Fifty-nine Parkinson’s disease patients and 54 controls were enrolled in this study.

Inclusion criteria were a clinical diagnosis of idiopathic PD according to the UK Parkinson’s Disease Society Brain Bank Diagnostic Criteria, Hoehn–Yahr stage I–IV; stable antiparkinsonian medication dose for 3 months; ability to speak and understand Lithuanian; and willingness to participate in the study [29]. Participants were excluded from the study if they had any of the following conditions: atypical Parkinsonism or other Parkinsonian disorders; other central nervous system disorders or somatic disorders that could influence dietary habits (such as food intolerance, diabetes mellitus, stomach ulcers, inflammatory bowel disease, or irritable bowel syndrome). Only subjects who reported stable dietary habits over the past 12 months and were not adhering to medically prescribed diets or structured nutritional interventions were included in the study. Additionally, individuals with psychiatric conditions (including psychosis, substance abuse, or significant depression) were excluded. Participants with moderate to severe cognitive impairment, as indicated by a Mini-Mental State Examination (MMSE) score of 24 points or less, were also excluded from the study.

Controls participants were recruited from individuals visiting the Neurology outpatient department for minor health issues, such as low back pain, radiculopathy, or peripheral mononeuropathies. These participants did not have any central nervous system disorders or other somatic conditions that could affect their dietary habits and nutritional intake.

This study was approved by the Vilnius Regional Bioethics Committee (Approval Number 2022/6-1434-910). All participants agreed to participate in the study, were informed of the study procedures, and provided written informed consent by signing the relevant written informed consent form.

### 2.2. Data Collection and Tools

All participants provided information about their social and demographic characteristics (age, gender, occupation, marital status, income, perceived financial status, smoking status, physical activity), as well as details about their health or disease status (comorbidities, PD duration, medication use). The age of PD onset was defined as the age at which the participant first experienced motor symptoms. Quantitative motor severity assessment was evaluated with the Movement Disorder Society—Unified Parkinson’s Disease Rating Scale—Part III (MDS-UPDRS III) and the Hoehn and Yahr scale [30,31]. Medications were compared following standard methods for calculating the daily Levodopa equivalent dose (LED) [32]. The Epworth Sleepiness Scale (ESS) was used to assess daytime sleepiness; 10 points or greater was set as a cutoff [33,34]. Parkinson’s Disease Sleep Scale-2 (PDSS-2) was used to assess sleep quality, using 18 points as a cut-off to determine poor sleep quality [35]. The Hospital Anxiety and Depression Scale (HADS) was used to assess anxiety and depression levels, with a cut-off score of ≥11 for clinically relevant depressed mood and ≥7 for clinically relevant anxiety [36,37,38]. The Parkinson’s Disease Fatigue Scale (PSF-16) was used for fatigue evaluation, with a cut-off score of ≥3.3 to indicate significant fatigue [39]. Cognitive functioning was assessed using the Montreal Cognitive Assessment (MoCA) [40]. The presence of constipation was assessed according to the Rome IV criteria [41]. The corresponding MDS-UPDRS Part I items were used to assess the presence of non-motor symptoms, including impulse-control disorder (item 1.6), pain (item 1.9), urinary dysfunction (item 1.10), and orthostatic hypotension (item 1.12). Parkinson’s Disease Quality of Life Questionnaire–39 (PDQ-39) was used to assess the quality of life of the PD participants [42].

Body weight was measured on a calibrated electronic scale with the individual wearing light indoor clothing and no shoes. Height was measured using a tape measure in the standing position without shoes. The Body Mass Index (BMI) was calculated. The World Health Organization criteria for obesity were used to classify BMI levels: underweight (BMI < 18.5), 153 healthy weight (BMI = 18.6–24.9), overweight (BMI = 25–29.9), and obesity (BMI > 30) [43].

Physical activity (PA) was assessed using a short version of the International Physical Activity Questionnaire (IPAQ). The recorded physical activity was quantified as metabolic equivalent minutes (MET) per week for each type of PA, and the overall MET was computed by summing these values. Subsequently, the data obtained were processed, adhering to the guidelines set by the IPAQ, and the total MET-min/week score was classified into three levels of low (<600 MET-minutes/week), moderate (600–3000 MET-minutes/week), and high (>3000 MET-minutes/week) physical activity [44].

The assessment of dietary intake for energy and nutrients was conducted using a 24-h dietary recall over two non-consecutive days. Data collection involved face-to-face interviews. An atlas of commonly consumed foods and their portion sizes was used to record the types and quantities of food each respondent had consumed over the previous 24 h. Two non-consecutive days were selected for the dietary recall because this is the minimum number of days required to accurately estimate an individual’s intake. Non-consecutive days were preferred to avoid potential correlations in food choices that might occur if consecutive days were assessed [45]. We excluded the assessment of alcohol intake from this study because an estimation of a person’s mean alcohol intake based only on two 24-h recalls is misleading, as alcohol intake can vary significantly between days.

The total average daily consumption of energy, macronutrients, and micronutrients was estimated using Alimenta nutritional software (version 4.2) that includes a comprehensive food composition database supplemented with information on typical foods and dishes commonly consumed in Lithuania. The total average daily consumption of macronutrients and micronutrients was calculated, as well as the percentage of total energy intake for such macronutrients as protein, fats, saturated fatty acids (SFAs), monounsaturated fatty acids (MUFAs), polyunsaturated fatty acids (PUFAs), carbohydrates, and total sugars.

Energy intake and consumption of macro- and micronutrients were compared between individuals with Parkinson’s Disease (PD) and control subjects. These values were also compared with the general nutrient recommended daily intakes (RDI) approved by the Minister of Health of the Republic of Lithuania, since PD-specific recommendations are lacking [46]. Since the mean age of our cohort was 67.03 ± 6.78 years, we used recommendations for individuals aged 65 and older for data analysis. The energy intake of the participants was compared with the average requirements of men and women aged ≥65 years, based on a range of energy intakes, using a physical activity level (PAL) of 1.4–1.8. Recommended daily intakes are listed in Supplementary Table S1.

### 2.3. Statistics

An a priori sample size calculation was performed using G*Power for Windows version 3.1 (Heinrich Heine University, Düsseldorf, Germany). The calculation was based on a two-tailed independent samples *t*-test, assuming a moderate effect size (Cohen’s d = 0.5), a significance level of *p* < 0.05, and a statistical power of 0.85. The estimated required sample size was 73 participants per group. Due to the exploratory nature of the study and feasibility constraints, a total of 113 participants (59 patients with Parkinson’s disease and 54 healthy controls) were included. In order to evaluate the adequacy of the achieved sample, we conducted a post hoc sensitivity analysis. The observed difference in the proportion of energy derived from carbohydrate between groups yielded a medium-to-large effect size (Cohen’s d = 0.75). At α = 0.05 and power = 0.85, this corresponds to a minimum requirement of 33 participants per group, confirming that the present study was adequately powered to detect clinically meaningful differences of this magnitude. All statistical analyses were conducted using SPSS version 26.0 (IBM Corp., Armonk, NY, USA) and R 4.5.2 (R Core Team, Vienna, Austria). The normality of data distribution was tested using the Shapiro–Wilk test. Normally distributed continuous variables are expressed as mean ± SD, and non-normally distributed continuous variables are expressed as median and interquartile range (IQR). Categorical variables are expressed as a percentage. Differences between the two groups were analyzed using a Student’s *t*-test for normally distributed variables and a Mann–Whitney U test for non-normally distributed continuous variables. A one-sample *t*-test was used to compare macro- and micronutrient intake to RDI. The Pearson and Spearman rank correlation coefficients were calculated to test the association between two variables with normal or non-normal distributions, respectively. Logistic and linear regression models were constructed to evaluate differences in dietary intake between the PD and controls, as well as within the PD cohort, after controlling for demographic and clinical variables. A *p*-value < 0.05 was considered statistically significant.

Given the exploratory nature of the study and the large number of comparisons, no formal multiple-comparison adjustment was applied. The results were interpreted with caution, considering effect sizes and clinical relevance in addition to statistical significance.

## 3. Results

### 3.1. Demographic and Disease-Related Characteristics

The participants’ average age at assessment was 67.03 ± 6.78 years, and 55.9% were men. There were no differences between the PD and control groups in age, gender, or educational duration. The subjects in the control group had a higher employment rate, with 35 (64.8%) controls employed, compared to 21 (35.6%) in the PD group (*p* = 0.002), and higher incomes, 38 (70.4%) and 24 (40.7%), respectively (*p* = 0.002). However, the subjective assessment of financial status did not differ between the groups (*p* = 0.077). Among PD patients, there were more never-smokers compared to the controls, 39 (66.1%) and 25 (46.3%), respectively (*p* = 0.041). PD patients more frequently had regular physical activity than controls, 23 (39.0%) and 12 (22.2%), respectively (*p* = 0.054). Most participants in both groups were either overweight (28 (47.5%) and 24 (44.5%), respectively) or obese (15 (25.4%) and 19 (35.2%), respectively). A detailed overview of the study population’s demographic characteristics is shown in Table 1.

The mean age at PD diagnosis was 59.58 ± 6.84 years, and the median disease duration was 6.00 (3–11) years. Most respondents (64.4%) had Hoehn–Yahr stage II; the MDS-UPDRS III score ranged from 10 to 55 points, with a mean of 29.98 ± 12.61. All participants were taking antiparkinsonian medications: 47 (79.8%) were on Levodopa, and 51 (86.4%) were on dopamine agonists. The median LED was 880 mg (532–1250 mg). According to MDS-UPDRS Part I, orthostatic hypotension was reported by 39 (66.1%), urinary disorders by 47 (79.7%), impulse control disorders by 12 (20.3%), and pain by 45 (76.3%). Olfactory disorder was reported by 32 (54.2%), and constipation was determined in 39 (66.1%) patients. Table 2 shows the disease-related characteristics of PD participants.

### 3.2. Macro- and Micronutrient Intake

**Figure 1:**
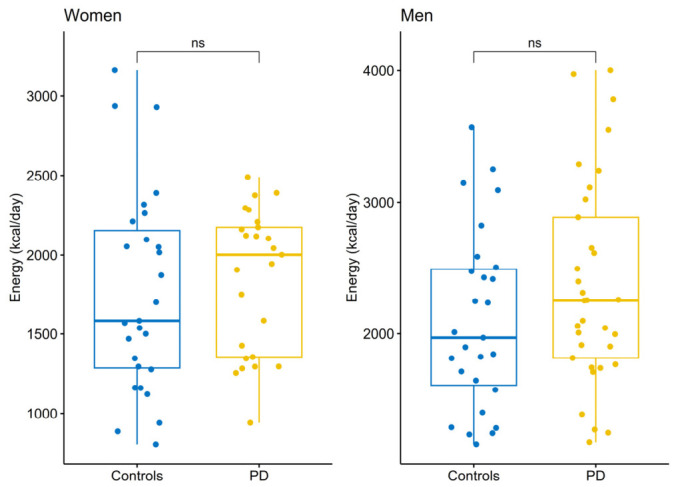
Total energy intake in the patients with Parkinson’s and the control group by gender. ns—not significant.

**Figure 2:**
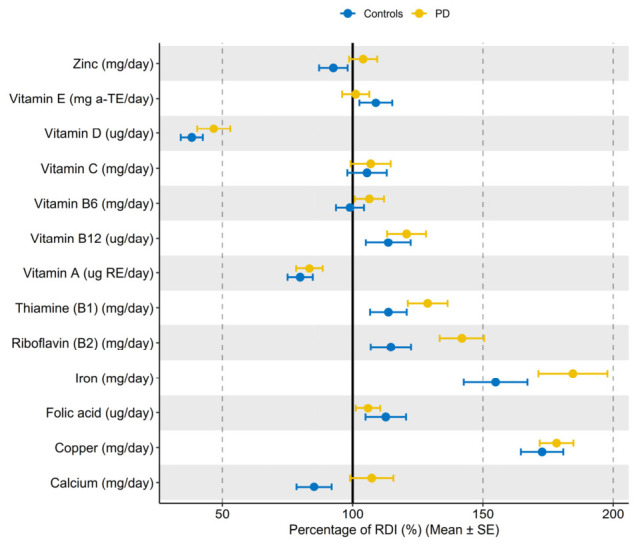
Respondents’ vitamin and mineral intake as a percentage of RDI. RDI—Recommended Daily Intake.

The mean daily energy intake among PD patients was 2363.88 ± 777.13 kcal/day for men and 2001.75 (1351.53–2192.28) kcal/day for women, which did not differ from the RDI and controls (Figure 1). PD patients consumed fewer dietary fibers (20.63 (15.38–27.53) g/day, *p* < 0.021), vitamin A (627.15 (458.1–789.81) μgRE/day, *p* < 0.001), and vitamin D (3.18 (1.97–5.13) μg/day, *p* < 0.001) compared to the RDI. PD patients had a higher total sugar-derived energy contribution than the RDI (11.31 (8.69–15.47) E%, *p* = 0.0121), while protein-, carbohydrate-, and fat-derived energy contributions were within the RDI range. However, less than half of PD patients reached the RDI for protein-, fat-, PUFA-, and MUFA-derived energy contribution, as well as for Vitamins A, D, E, C, Calcium, and Zinc (Table 3 and Figure 2). Compared to controls, PD patients consumed more dietary fibers (20.63 (15.38–27.53) g/day vs. 16.9 (12.35–23.24) g/day, *p* = 0.029), plant protein (41.22 (24.79–54.13) g/day vs. 28.07 (18.2–47.8) g/day, *p* = 0.019), and total carbohydrates (261.42 (189.7–333.78) g/day vs. 196.87 (153.13–271.87) g/day, *p* = 0.002). They had higher carbohydrate-derived energy contribution (50.3 ± 7.01 E% vs. 44.3 ± 8.94 E%, *p* < 0.001) while fat-derived energy contribution was lower (23.54 ± 6.33 E% vs. 26.94 ± 7.82 E%, *p* = 0.013). Statistical significance between the groups persisted after controlling for age, gender, and BMI for carbohydrates (*p* = 0.007), as well as for fat- (*p* = 0.009), PUFA- (*p* = 0.015), MUFA- (*p* = 0.011), and carbohydrate-derived energy contribution (*p* < 0.001), but not for dietary fiber (*p* = 0.06). Detailed results on dietary intake in PD compared with controls and RDI are presented in Table 3. Although the protein-derived energy contribution was within the recommended range in both PD and control groups, with no significant differences between groups, PD patients consumed more protein per kilogram of body weight (1.01 (0.76–1.38) g/kg/day) than controls (0.81 (0.66–1.20 g/kg/day), *p* = 0.019, and intake in the PD group did not differ from the recommended daily intake for older adults (1.0 g/kg/day), *p* = 0.319.

In the PD group, women had lower energy intake (2001.75 (1351.53–2192.28) kcal/day vs. 2363.88 ± 777.13 kcal/day, *p* = 0.028), fat intake (46.74 ± 17.26 g/day vs. 61.37 ± 19.56 g/day, *p* = 0.004), including all components (SFAs, MUFAs, PUFAs and cholesterol), and dietary fiber intake (19.3 ± 7.92 g/day vs. 21.72 (15.96–32.49) g/day, *p* = 0.039) compared to men. Women, but not men, consumed less than 300 mg/d of cholesterol, as recommended by the RDI (219.7 (144.38–315.51) mg/day vs. 313.28 ± 121.68 mg/day, *p* = 0.005). Men had a higher total sugar-derived energy contribution (11.82 ± 4.98 E%, *p* = 0.044), and consumed less vitamin A (709.81 ± 311.93 μgRE/day, *p* = 0.001), while women consumed less dietary fiber (19.3 ± 7.92 g/day, *p* = 0.001) compared to RDI and compared to men (*p* = 0.039). Vitamin D consumption was lower than RDA in both groups (3.55 (2.51–4.65) μg/day, *p* < 0.001 for men and 2.48 (1.76–6.1) μg/day, *p* < 0.001 for women). A detailed overview of PD dietary intake by gender is shown in Supplementary Table S2.

### 3.3. Dietary Intake Associations with Parkinson’s Disease Characteristics

We found no significant correlations between dietary intake of energy, micronutrients, and macronutrients, and age, age at motor symptom onset, disease duration, PDQ-39 total score, LED, dopamine agonists’ LED, MDS-UPDRS total score, or MDS-UPDRS motor part score.

We compared dietary intake between PD patients with and without specific non-motor symptoms. We found no difference between those with excessive daytime slippiness, sleep problems, anxiety, impulse control disorder, orthostatic hypotension, urinary dysfunction, constipation, or hyposmia and those without. However, those with cognitive impairment consumed less dietary fiber than those without (19.91 ± 7.41 g/day vs. 25.26 ± 12.1 g/day, *p* = 0.045). The difference persisted after controlling for age, sex, and disease duration (β = −5.658, r2 = 0.11, *p* = 0.035). PD patients who experienced fatigue consumed more fats (58.79 ± 19.62 g/day vs. 47.39 ± 18.43, *p* = 0.034) and PUFAs (5.55 ± 1.97 E% vs. 4.39 ± 1.28 E%, *p* = 0.008). The difference persisted after controlling for age, sex, and disease duration (β = 11.702, r2 = 0.157, *p* = 0.047, and β = 1.347, r2 = 0.067, *p* = 0.018, respectively). PD patients who had a depressive mood had a lower carbohydrate-derived energy contribution (49.42 ± 7.36 E% vs. 54.12 ± 3.25 E%, *p* = 0.002) and lower total carbohydrate (262.35 ± 105.15 g/day vs. 377.92 ± 115.58 g/day, *p* = 0.041) and dietary fiber consumption (20.97 ± 9.4 g/day vs. 29.91 ± 11.67 g/day, *p* = 0.034) compared to those without depressive mood, even after controlling for age, sex, and disease duration (β = −5.686, r2 = 0.09, *p* = 0.016, and β = −82.25, r2 = 0.057, *p* = 0.030, and β = −8.494, r2 = 0.139, *p* = 0.013 respectively). Also, patients with depressive mood consumed less plant protein (40.89 ± 24.57 g/day vs. 66.97 ± 36.78 g/day, *p* = 0.045; controlling for age, sex, and disease duration, β = −24.598, r2 = 0.103, *p* = 0.011), vitamin B1 (1.38 ± 0.63 mg/day vs. 1.98 ± 0.83 mg/day, *p* = 0.044; controlling for age, sex, and disease duration, β = −0.543, r2 = 0.092, *p* = 0.021), vitamin B2 (1.72 ± 0.83 mg/day vs. 2.49 ± 0.91 mg/day, *p* = 0.022; controlling for age, sex, and disease duration, β = −0.7, r2 = 0.086, *p* = 0.019), calcium (868.89 ± 302.88 mg/day vs. 1388.47 ± 597.67 mg/day, *p* = 0.037; controlling for age, sex, and disease duration, β = −480.801, r2 = 0.085, *p* = 0.013), iron (16.94 ± 9.6 mg/day vs. 25.06 ± 10.39, *p* = 0.032; controlling for age, sex, and disease duration, β = −7.948, r2 = 0.055, *p* = 0.022) and copper (1.71 ± 0.47 mg/day vs. 2.09 ± 0.51 mg/day, *p* = 0.044; controlling for age, sex, and disease duration, β = −0.424, r2 = 0.127, *p* = 0.010) compared to those without. PD patients with orthostatic hypotension consumed less vitamin B12 than those without (2.88 ± 1.5 μg/day vs. 4.00 ± 1.72 μg/day, *p* = 0.013, controlling for age, sex, and disease duration, β = −1.148, r2 = 0.051, *p* = 0.022).

## 4. Discussion

The results of this study provide novel and clinically important insights into the dietary habits of Lithuanian PD patients.

The energy intake of the participants was similar to the average requirements for men and women aged > 65 years old based on PAL 1.4–1.8, and did not differ from controls. Data on energy intake among PD patients is conflicting. Similar results were found by Marczewska et al., Palavra et al., and Ådén et al. [18,47,48]. However, Barichella et al. and Dunk et al. found that energy intake was significantly higher in PD compared to controls [49,50]. In the present study, women had lower energy intake than men. However, in contrast, Baert et al. found no difference in energy intake between men and women [51]. According to a study on energy, macronutrient, and micronutrient intake among the adult population in Lithuania, conducted from 2019 to 2020, women also had lower energy consumption [6]. Inconsistencies in results may be attributed to the fact that energy requirements and homeostasis can vary across PD stages. In the early stage of disease, non-motor symptoms such as smell and taste disorders, mood changes, and gastrointestinal dysfunction have been linked to decreased food intake in PD patients. As diseases progress, motor symptoms such as tremor, rigidity, and dyskinesia emerge, which may contribute to increased energy expenditure and requirements [12].

We found that carbohydrate-derived energy consumption in patients with PD was higher than in controls, and PD patients had higher total sugar-derived energy consumption compared to the RDI. However, according to the latest evaluation of dietary intake in the Lithuanian adult population, conducted in 2019–2020, energy intake from fats exceeded the recommended norms, while energy intake from carbohydrate was below the lower range [6]. This discrepancy could be attributed to the small sample size and age difference. The Lithuanian population study examined dietary intake among adults aged 18 to 65, whereas our cohort’s average age was 65 years or older. Nevertheless, increased carbohydrate and sugar consumption is consistently reported in patients with PD [17,18,47,49,50]. Several mechanisms can explain increased sugar consumption. It may serve as a compensatory mechanism for disease-related dopamine loss, as sugar may increase brain dopamine through insulin and alter brain activity in reward-processing regions [52]. Higher sugar consumption can also be associated with the use of dopamine agonists that are linked to impulse control disorders and binge eating [17,53]. However, there was no difference in carbohydrate or total sugar intake between individuals with and without impulse control disorders, nor was there a correlation between carbohydrate and sugar intake and dopamine agonist intake in our PD cohort. Mood disorders, like anxiety and depression, as well as smell and taste disturbances, are common in patients with PD [54]. Consumption of carbohydrate- and sugar-rich foods can transiently enhance serotonergic neurotransmission and improve mood, potentially reinforcing sweet food intake as a form of emotional self-regulation [55]. Additionally, decreased taste function can lead to increased sugary food-seeking behavior [18].

In addition to behavioral drivers of carbohydrate and sugar consumption in PD, metabolic factors have emerged as relevant aspects of disease risk and progression. Some studies demonstrate that increased carbohydrate intake in the past is associated with an increased risk of PD [56]. Epidemiological and experimental studies indicate that chronic hyperglycemia, insulin resistance, and type 2 diabetes mellitus (T2DM) are associated with an increased risk of PD [57,58]. Metabolic dysfunction may promote neurodegenerative processes through impaired glucose utilization, enhanced glycation, and neuroinflammation, linking high carbohydrate and sugar intake with systemic insulin dysregulation that could contribute to PD pathophysiology [58]. Notably, the glucagon-like peptide-1 (GLP-1) receptor, a key regulator of glucose homeostasis, has been increasingly investigated for its potential neuroprotective effect in PD. GLP-1 receptor agonists, initially developed for T2DM, have demonstrated beneficial effects on insulin sensitivity, mitochondrial function, neuroinflammation, and neuronal survival in both preclinical models and early clinical studies, suggesting that modulation of this pathway may ameliorate metabolic and neurodegenerative processes relevant to PD [59].

High sugar intake may also affect the gut–brain axis by altering gut microbiota composition, leading to increased intestinal permeability and neuroinflammation, both of which are implicated in PD pathophysiology [60].

In the present study, we found that patients with PD consumed more plant protein than the control group, while their total protein intake did not differ from that of the control group or the RDI. Similar results were found by Marczewska et al. [48]. Lower protein intake in PD patients has not been consistently observed, despite a low-protein redistribution diet being recommended for patients with advanced disease to improve Levodopa absorption and alleviate motor fluctuations [19,49,50,51]. However, some authors found diminished protein intake among patients with PD [17,18,47]. Higher plant protein consumption may be attributed to increased fruit and vegetable consumption in patients with PD, as reported in previous studies [61]. Patients with PD frequently report gastrointestinal dysfunctions such as delayed gastric emptying, gastroparesis, and constipation [15]. Accordingly, these patients are often advised to increase their intake of fiber-rich foods, such as fruits, vegetables, and whole grains, as dietary fiber can help alleviate constipation and increase Levodopa bioavailability [51,62,63,64]. According to our results, 66.1% of patients in the PD cohort had constipation. Notably, our study found that patients with PD consumed more dietary fiber compared to the control group, with men consuming more than women. However, dietary fiber intake was below the RDI in both groups, consistent with the low dietary fiber intake in the Lithuanian population. However, men, compared with women, and older adults, compared with the youngest adults, had higher dietary fiber intake in the general Lithuanian population [6]. Increased dietary fiber intake in the PD population has also been reported previously [48,49,51]. However, other studies found decreased intake or no difference compared to controls [17,18,47,50].

Our study results indicated that the intake of the evaluated minerals and vitamins was similar between the PD and control groups and generally met the RDI. However, the intake of vitamin A and vitamin D was below the RDI in both groups. It is important to note that potential supplement intake was not taken into account. According to the most recent survey on dietary intake of the Lithuanian population, the consumption of most vitamins and minerals was found to be insufficient [6]. Low vitamin D intake in patients with PD has also been reported in previous studies, highlighting the potential need for screening for vitamin D deficiency and timely supplementation [49,50,51,65]. The prevalence of PD increases with age, particularly among those aged 60 years or older; meanwhile, the risk of vitamin D deficiency increases among older people, as well as among residents of northern latitudes, such as Lithuania [66,67]. Individuals with PD have increased fall risk because of impaired balance, gait disturbances, cognitive dysfunction, and autonomic failure, as well as increased risk of osteoporosis and bone fractures [68,69]. Vitamin D supplementation has not been widely evaluated in people with PD for bone and muscular health. However, according to a recent review of osteoporosis management in PD, vitamin D supplementation slows the decline in bone mineral density, improves muscle strength, reduces falls, and reduces fracture risk [70].

We found no correlation between dietary intake and disease-related characteristics, like age, age at motor symptom onset, disease duration, quality of life scale, total and dopamine agonist-derived LED, or disease severity measured by the MDS-UPDRS part III scale. However, these results should be interpreted cautiously due to the small sample size for correlational analysis. In contrast, Palavra et al. reported that a worse quality of life (as measured by the PDQ-39 score) correlated with higher total free sugar intake and lower alcohol consumption. Additionally, increased total sugar consumption was associated with a greater daily Levodopa dose requirement and a greater burden of overall non-motor symptoms (measured by the Non-motor Symptoms Scale (NMSS)), notably worse constipation and upper gastrointestinal dysfunction [18]. According to Kwon et al.’s results, PD patients who were younger at diagnosis or had longer disease duration tended to consume greater amounts of total sugars, added sugars, and trans fats. They also found an association between higher doses of dopamine agonists and increased consumption of sugar and trans fats [17]. The correlation between total protein intake and daily Levodopa dosage has also been previously reported [48].

We found some dietary intake differences in PD patients with cognitive impairment, fatigue, depressive mood, and orthostatic hypotension. Nonetheless, the cross-sectional nature of the study limits the ability to determine the directionality of the observed associations. However, some findings are supported by previous studies. Higher dietary fiber intake is reported to be associated with better cognitive function in elderly adults [71,72]. The potential mechanism underlying the favorable effect of dietary fiber on cognition involves modulation of the gut–brain axis through alterations in gut microbiota composition and increased production of short-chain fatty acids, decreased neuroinflammation, and promotion of neuroprotective factors synthesis [73,74].

In the present study, PD patients with orthostatic hypotension had lower vitamin B12 intake than those without orthostatic hypotension. Vitamin B12 deficiency is a well-known cause of polyneuropathy and can lead to autonomic nerve damage [75,76]. Some case reports present vitamin B12 deficiency causing autonomic neuropathy with orthostatic hypotension, which resolved after correction of the deficiency [77]. So, vitamin B12 deficiency could be considered as an exacerbating factor of orthostatic hypotension in patients with PD.

We found that PD patients with depressive mood consumed fewer carbohydrates, dietary fiber, plant protein, and certain vitamins and minerals. In contrast, Palavra et al. reported that PD patients who were depressed consumed more total sugars and less alcohol compared to those who were not depressed [18].

Although no association was found between overall dietary intake and disease severity, specific relationships between dietary components and non-motor symptoms suggest that nutrition may modulate these symptoms. Given the multifactorial nature of non-motor symptoms in PD, dietary factors may represent a modifiable target, although the cross-sectional data do not allow for establishing causal relationships. Moreover, there is some evidence that specific dietary patterns are associated with non-motor symptoms in individuals with PD. For instance, the Dietary Approach to Stop Hypertension (DASH) can improve brain function and slow cognitive impairment, and the Mediterranean dietary pattern can improve constipation and cognitive function and is associated with fewer or less severe non-motor symptoms, including cognitive impairment, depression, and sleep disturbances, in patients with PD [78,79,80,81,82].

Dietary macronutrients affect the brain and cognition through a variety of pathways, including glucose and insulin metabolism, neurotransmitter effects, and cerebral oxidation and inflammation [83]. There is evidence that foods high in sugar and carbohydrate can temporarily boost serotonergic transmission, which may reinforce the consumption of sweet foods as a method of emotional self-regulation [55]. Conversely, individuals with depressive disorders often experience a reduced appetite, which may lead to diminished nutrient intake and specific food choices, resulting in poorer diet quality [84,85,86]. Notably, in our cohort, PD patients with depressive moods consumed fewer carbohydrates, dietary fiber, plant protein, vitamin B1, vitamin B2, calcium, iron, and copper. However, their total energy intake was similar to that of patients without depressive moods.

Although we did not observe an association between dietary intake and disease duration, previous studies suggest that nutritional status in PD may change over time. Early-stage disease is often characterized by non-motor symptoms, such as olfactory and gustatory disturbances, mood changes, and gastrointestinal disturbances, which may contribute to decreased nutrient intake. In contrast, later stages are associated with worsening motor symptoms (e.g., tremor, rigidity, and dyskinesia), as well as mood and psychiatric symptoms, which may increase energy demands and lead to behavioral changes that influence dietary intake [12]. Antiparkinsonian medication intake can also influence patients’ nutritional habits [16]. Although deficiencies in micronutrients such as vitamin D, vitamin B12, and folate have been reported in PD, most evidence comes from cross-sectional studies, and longitudinal data on how nutritional status evolves with disease progression remain limited [23,87].

On the other hand, some longitudinal and observational studies indicate that healthier dietary patterns (e.g., higher intake of fruits, vegetables, and fish) are associated with slower PD progression [88]. In contrast, processed and high-sugar foods may be associated with a faster progression [88]. However, large-scale longitudinal datasets specifically addressing detailed dietary intake in PD remain limited, and most available studies are short-term or observational. So, longitudinal data are essential for understanding how dietary intake evolves with disease progression in PD.

The main limitations of the present study include a cross-sectional design, a small sample size, and potential selection bias due to participants’ recruitment from a single center. Although there are only 3 tertiary movement disorder centers in Lithuania, patients from our center were considered representative of Lithuanian PD patients, as they come from different regions of the country. Additionally, patients who consent to participate in the survey may be more interested in healthy dietary habits and may have greater adherence to a healthier diet than other patients with PD.

Another potential limitation is that the control group consisted of individuals with minor neurological conditions (e.g., low back pain, radiculopathy). While such conditions can sometimes be associated with altered nutritional intake or lifestyle changes, we sought to mitigate this by only including participants who reported stable dietary habits over the past 12 months and no adherence to prescribed medical diets [89,90].

Another potential limitation of our study is a recall bias. PD patients may experience cognitive impairment leading to questionable recall of dietary intake. To mitigate this potential bias, patients with MMSE scores ≤24 points were excluded from the study. However, using the MOCA, we found that, despite MMSE results, about 50% of patients had some cognitive dysfunction.

In addition, assessment bias associated with the method used to evaluate dietary intake should be acknowledged. The dietary recalls may be sensitive to misreporting, seasonal variation in dietary intake, and the risk of under- and overreporting. Additionally, we utilized data from only two non-consecutive 24-h dietary recalls, although longer recording periods are recommended in studies with small sample sizes. A dietary recall of two non-consecutive days was chosen to minimize participant dropout. To mitigate potential biases associated with 24-h dietary recalls, recalls were conducted on both weekdays and weekends, with a gap of 3 to 6 months between each. Furthermore, we used an atlas of commonly consumed foods and their portion sizes to provide more accurate estimates.

Dietary habits may vary across seasons, potentially influencing the accuracy of our dietary assessments [91,92]. This study is cross-sectional, and each participant’s dietary intake was evaluated using two-day 24-h recalls averaged into a single estimate. Unfortunately, the fact that enrollment dates were not recorded as a variable in the dataset precludes a formal statistical adjustment for season. Given the concurrent recruitment of both participants with PD and controls throughout the study period from January 2023 to January 2025, we believe that any seasonal influences on dietary habits will be distributed approximately equally between the two groups. This suggests that while seasonal variation in diet is a potential limitation, it is unlikely to introduce systematic bias in our between-group comparisons. However, future studies should incorporate seasonality as a variable and use more frequent dietary assessments to capture fluctuations in dietary habits over time.

Also, it is important to note that dietary intake values obtained from 24-h recalls represent estimates rather than precise measures of habitual intake; however, they are appropriate for group-level comparisons. Residual variability cannot be fully excluded.

Although sarcopenia is common in PD patients and is associated with poor nutritional status and functional outcomes, this study did not include direct assessments of muscle mass, strength, or physical performance, which limits our ability to evaluate sarcopenia and its relationship with dietary intake [13,93]. Future studies should incorporate standardized sarcopenia assessments to elucidate the association among nutrition, muscle health, and clinical aspects of PD.

Additionally, the comparability of our results to other studies is limited due to the different dietary assessment tools utilized. Dietary habits vary significantly across ethnicities, limiting the generalizability of our findings [28].

Nonetheless, the study provides valuable insights into the dietary intake of Lithuanian patients with PD, being the only dietary data to our knowledge for a Lithuanian PD population. The strength of this study is that dietary data were collected under the supervision of a trained investigator, and PD diagnosis and disease-related scale evaluation were performed by a neurologist experienced in movement disorders.

## 5. Conclusions

In conclusion, we found that most subjects in the PD group, as in the control group, were overweight or obese. Also, we found that patients with PD had higher total sugar-derived energy consumption and lower dietary fiber and vitamin D intake than the RDI. In addition, we revealed differences in dietary intake among PD patients with certain non-motor symptoms. These findings have important clinical implications. They highlight the need for routine nutritional assessment and targeted, personalized dietary counseling as part of comprehensive PD management, which should address potentially modifiable factors to improve overall health and specific non-motor symptoms and prevent metabolic dysregulation. Interventions to increase dietary fiber intake may improve gastrointestinal function. Furthermore, given the consistently low vitamin D intake, health care specialists should consider screening for deficiency and appropriate supplementation, particularly in older individuals with increased risk of vitamin D deficiency, osteoporosis, and bone fractures. Additionally, dietary habits may differ among patients with specific non-motor symptoms, supporting a more individualized, symptom-oriented nutritional approach. This research should encourage further longitudinal studies of PD patients in Lithuania with larger cohorts, and the inclusion of body composition measures is warranted to elucidate further the role of nutrition in disease progression and clinical outcomes in PD.

## Figures and Tables

**Table 1:** Demographic characteristics of the study population.

Variable	PD (*n* = 59)	Controls (*n* = 54)	*p* Value
Age, years	67.0 ± 6.8	65.7 ± 7.4	0.329
Male, *n* (%)	33 (55.9)	27 (50.0)	0.528
Marital status, *n* (%)			0.849
Married/partner	45 (76.3)	42 (77.8)	
Single/divorced/widowed	14 (23.7)	12 (22.2)	
Education, years	16.0 (13–17)	14.5 (13–16)	0.072
Education level, *n* (%)			0.015 *
Secondary	6 (10.2)	4 (7.4)	
College	14 (23.7)	27 (50.0)	
University	39 (66.1)	23 (42.6)	
Residency, *n* (%)			0.476
City	56 (94.9)	49 (90.7)	
Rural	3 (5.1)	5 (9.3)	
Employed, *n* (%)	21 (35.6)	35 (64.8)	0.002 *
Financial status, *n* (%)			0.077
Good	12 (20.3)	21 (38.9)	
Average	44 (74.6)	32 (59.3)	
Difficult	3 (5.1)	1 (1.9)	
Income ≥1000 €/month, *n* (%)	24 (40.7)	38 (70.4)	0.002 *
Smoking status, *n* (%)			0.041 *
Never	39 (66.1)	25 (46.3)	
Former	13 (22.0)	13 (24.1)	
Current	7 (11.9)	16 (29.6)	
Regular physical activity, *n* (%)	23 (39.0)	12 (22.2)	0.054
Physical activity frequency, *n* (%)			0.008 *
Daily	15 (65.2)	1 (8.3)	
2–3/week	7 (30.4)	8 (66.7)	
1/week	1 (4.4)	3 (25.0)	
IPAQ level, *n* (%)			0.285
Low	5 (8.5)	4 (7.4)	
Moderate	31 (52.5)	21 (38.9)	
High	23 (39.0)	29 (53.7)	
BMI, kg/m^2^	27.5 ± 4.3	28.4 ± 3.9	0.226
BMI category, *n* (%)			0.537
Underweight	1 (1.7)	0	
Normal	15 (25.4)	11 (20.4)	
Overweight	28 (47.5)	24 (44.5)	
Obesity	15 (25.4)	19 (35.2)	

Values are given as mean ± standard deviation, median (interquartile range), or number (percentage). BMI, body mass index; IPAQ, international physical activity questionnaire; PD, Parkinson’s disease. * Statistical significance, *p* < 0.05.

**Table 2:** Disease-related characteristics of the PD group.

Variable	PD (*n* = 59)
Age at diagnosis, years	59.6 ± 6.8
Disease duration, years	6.0 (3–11)
MDS-UPDRS III	30.0 ± 12.6
Hoehn–Yahr stage, *n* (%)	
Stage I	5 (8.5)
Stage II	38 (64.4)
Stage III	14 (23.7)
Stage IV	2 (3.4)
Non-motor symptoms, *n* (%)	
Cognitive impairment	30 (50.8)
Daytime sleepiness	13 (22.0)
Sleep problems	23 (39.0)
Fatigue	20 (33.9)
Anxiety	20 (33.9)
Depression	11 (18.6)
Constipation	39 (66.1)
Orthostatic hypotension	39 (66.1)
Urinary dysfunction	47 (79.7)
Pain	45 (76.3)
Impulse control disorders	12 (20.3)
Hyposmia	32 (54.2)
PD medication	
Number of medications	3 (3–4)
LED, mg	880 (532–1250)
Dopamine agonist LED, mg	240 (120–320)
Oral levodopa use, *n* (%)	47 (79.7)
Dopamine agonists use, *n* (%)	51 (86.4)
Amantadine use, *n* (%)	30 (50.8)
MAO-B inhibitors use, *n* (%)	51 (86.4)
COMT inhibitors use, *n* (%)	17 (28.8)
Comorbidities, *n* (%)	
Hypertension	30 (58.8)
Cardiovascular disease	3 (5.1)
Osteoporosis	3 (5.1)
Hypothyroidism	4 (6.8)
Dyslipidemia	17 (28.8)
Gout	4 (6.8)
PDQ-39 summary index	20.7 (7.4–34.6)

Values are given as mean ± standard deviation, median (interquartile range), or number (percentage). MDS-UPDRS, Movement Disorders Society Unified Parkinson’s Disease Rating Scale, PD, Parkinson’s disease; LED, Levodopa equivalent dose; MAO-B, monoamine oxidase B; COMT, catechol-O-methyltransferase; PDQ-39, Parkinson’s Disease Questionnaire-39.

**Table 3:** Dietary intake of Parkinson’s disease patients compared to controls and the recommended daily intake.

Nutrient	PD (*n* = 59)	RDI (%) ^	Controls (*n* = 54)	RDI (%) ^	*p* Value ^§^	RDI	*p*-Value #
Energy, kcal (men)	2363.9 ± 777.1	27.3	2100.8 ± 677.4	25.9	0.167	2030–2670	0.748
Energy, kcal (women)	2001.8 (1351.5–2192.3)	34.6	1765.9 ± 639.0	22.2	0.374	1690–2170	0.708
Protein, E%	15.9 ± 3.6	37.3	15.9 ± 2.9	51.9	0.998	15–20	0.001 *
Fat, E%	23.5 ± 6.3	35.6	26.9 ± 7.8	38.9	0.013 *	25–35	0.082
SFAs, E%	8.6 (7.5–10.5)	66.1	9.5 ± 3.1	64.8	0.387	<10	0.004 *
MUFAs, E%	8.8 (6.9–11.5)	20.3	11.1 ± 3.7	31.5	0.005 *	10–14	0.064
PUFAs, E%	5.2 ± 1.8	30.5	5.6 (4.3–7.4)	31.5	0.109	6–10	<0.001 **
Carbohydrates, E%	50.3 ± 7.0	69.5	44.3 ± 8.9	46.3	<0.001 **	45–60	0.019 *
Total sugars, E%	11.3 (8.7–15.5)	37.3	11.7 ± 4.7	42.6	0.843	<10	0.012 *
Protein, g	78.6 (63.2–94.9)	NA	71.7 (55.0–98.7)	NA	0.094	–	NA
Protein, g/kg/day	1.01 (0.76–1.38)	52.5	0.81 (0.66–1.20)	33.3	0.019 *	1.0	0.319
Animal protein, g	39.7 ± 15.1	NA	40.8 ± 15.8	NA	0.700		NA
Plant protein, g	41.2 (24.8–54.1)	NA	28.1 (18.2–47.8)	NA	0.019 *	–	NA
Fat, g	54.9 ± 19.8	NA	52.5 (42.4–66.1)	NA	0.852	–	NA
SFAs, g	21.1 (16.0–24.3)	NA	19.2 (14.8–24.3)	NA	0.344	–	NA
MUFAs, g	18.9 (14.7–27.2)	NA	23.1 ± 9.6	NA	0.330	–	NA
PUFAs, g	11.3 (7.8–15.0)	NA	11.7 (8.2–15.8)	NA	0.583	–	NA
Cholesterol, mg	262.7 (186.0–349.8)	64.4	273.9 (203.8–378.3)	59.3	0.631	<300	0.072
Carbohydrates, g	261.4 (189.7–333.8)	NA	196.9 (153.1–271.9)	NA	0.002 *	–	NA
Total sugars, g	60.3 (37.9–80.2)	NA	49.9 (38.1–68.6)	NA	0.131	–	NA
Dietary fiber, g	20.6 (15.4–27.5)	35.6	16.9 (12.4–23.2)	22.2	0.029 *	25–35	0.021 *
Calcium, mg	842.4 (553.0–1094.2)	45.8	650.3 (371.6–1047.7)	37.0	0.055	900	0.709
Iron, mg	14.4 (10.1–23.9)	78.0	12.1 (9.2–20.8)	70.4	0.091	10	<0.001 **
Copper, mg	1.78 ± 0.50	98.3	1.73 ± 0.60	87.0	0.595	1.0	<0.001 **
Zinc, mg	10.0 (8.1–12.3)	49.2	9.3 ± 4.0	37.0	0.162	10	0.865
Vitamin A, μg RE	627.2 (458.1–789.8)	28.8	595.6 (399.8–798.1)	24.1	0.579	800	<0.001 **
Vitamin D, μg	3.2 (2.0–5.1)	10.2	2.7 (1.9–4.6)	3.7	0.433	10	<0.001 **
Vitamin E, mg α-TE	12.1 ± 4.8	49.2	13.1 ± 5.6	53.7	0.347	11	0.074
Vitamin B1, mg	1.39 (1.08–1.67)	69.5	1.14 (0.81–1.86)	48.1	0.131	1.1	<0.001 **
Vitamin B2, mg	1.67 (1.21–2.14)	69.5	1.26 (0.95–1.97)	51.9	0.013 *	1.3	<0.001 **
Folic acid, μg	202.5 (158.9–234.2)	52.5	198.1 (142.7–289.8)	48.1	0.870	200	0.731
Vitamin B6, mg	1.70 ± 0.69	54.2	1.58 ± 0.63	46.3	0.341	1.4	0.001 *
Vitamin B12, μg	3.62 ± 1.72	59.3	3.50 (2.01–4.40)	59.3	0.403	3.0	0.008 *
Vitamin C, mg	73.7 (54.9–101.7)	45.8	81.9 (45.2–111.5)	53.7	0.947	80	0.818

Values are given as mean ± standard deviation, median (interquartile range). The bold values indicate clinical significance. ^§^ *p*-value for difference between PD and controls, # *p*-value for difference between PD and RDI, ^ percent achieving RDI. MUFAs, monounsaturated fatty acids; NA, not applicable; PUFAs, polyunsaturated fatty acids; PD, Parkinson’s disease; SFAs, saturated fatty acids; RDI, recommended daily intake; * statistical significance, *p* < 0.05; ** statistical significance, *p* < 0.001.

## Data Availability

The original contributions presented in this study are included in the article and Supplementary Materials. Further inquiries can be directed to the corresponding author.

## Supplementary Materials

The following supporting information can be downloaded at: https://www.mdpi.com/article/10.3390/nu18081302/s1, Table S1: Recommended daily intakes (RDI) for Lithuanian adults aged > 65 years, Table S2: Dietary intake of the PD group by gender compared to the RDI.

## Abbreviations

The following abbreviations are used in this manuscript:

PD	Parkinson’s Disease
RDI	Recommended Daily Intake
SFAs	Saturated Fatty Acids
PUFAs	Polyunsaturated Fatty Acids
MUFAs	Monounsaturated Fatty Acids
BMI	Body Mass Index
MMSE	Mini-Mental State Examination
MDS-UPDRS	Movement Disorder Society—Unified Parkinson’s Disease Rating Scale
LED	Levodopa equivalent dose
ESS	Epworth Sleepiness Scale
PSS-2	Parkinson’s Disease Sleep Scale-2
HADS	Hospital Anxiety and Depression Scale
PSF-16	Parkinson’s Disease Fatigue Scale
MoCA	Montreal Cognitive Assessment
PDQ-39	Parkinson’s Disease Quality of Life Questionnaire-39
PA	Physical activity
IPAQ	International Physical Activity Questionnaire
MET	Metabolic Equivalent Minutes
PAL	Physical Activity Level
IQR	Interquartile Range
T2DM	Type 2 Diabetes Mellitus
GLP-1	Glucagon-like peptide-1
