# Supplementary material for: Dietary Intake of Patients with Parkinson’s Disease in Lithuania
Source: Nutrients. 2026 Apr 20;18(8):1302. doi: 10.3390/nu18081302 (PMC13118462; doi:10.3390/nu18081302)
Supplement: Supplementary file 1 [file nutrients-18-01302-s001.zip › nutrients-4211434-supplementary.pdf]

Dietary Intake of Patients with Parkinson's Disease in Lithuania

Jevgenija Guk, Rūta Kaladytė Lokominienė, Anatolij Nečiporenko, Roma Bartkevičiūtė, Albertas Barzda, Dalius Jatužis

Table S1. Recommended daily intakes (RDI) for Lithuanian adults aged >65 years.

Nutrient	Men	Women
**Energy, kcal (PAL 1.4–1.8)**	2030–2670	1690–2170
**Macronutrients**		
Protein, E%	10–20	10–20
Fat, E%	25–35	25–35
Saturated fat, E%	<10	<10
Carbohydrate, E%	45–60	45–60
Total sugars, E%	<10	<10
Dietary fiber, g/day	25–35	25–35
**Vitamins**		
Vitamin A, µg RE/day	900	700
Vitamin D, µg/day	10	10
Vitamin E, mg α-TE/day	12	10
Vitamin B1 (Thiamine), mg/day	1.2	1.0
Vitamin B2 (Riboflavin), mg/day	1.4	1.2
Vitamin B6, mg/day	1.6	1.2
Vitamin B12, µg/day	3.0	3.0
Folic acid, µg/day	200	200
Vitamin C, mg/day	80	80
**Minerals**		
Calcium, mg/day	900	900
Copper, mg/day	1.0	1.0
Iron, mg/day	10	10
Zinc, mg/day	10	10

PAL, Physical Activity Level; RDI, Recommended Daily Intake

Table S2. Dietary intake of the PD group by gender compared to the RDI.

Nutrient	Men (n=33)			Women (n=26)			P-value#
	Value	RDI	p-value	Value	RDI	P-value	
Energy, kcal	2363.9 ± 777.1	2030–2670	0.748	2001.8 (1351.5–2192.3)	1690–2170	0.708	0.028*
Protein, E%	16.0 ± 4.0	15–20	0.173	15.9 ± 3.2	15–20	0.015*	0.918
Fat, E%	24.5 ± 6.8	25–35	0.646	22.4 ± 5.6	25–35	0.024*	0.206
SFA, E%	9.3 ± 2.8	<10	0.183	8.8 ± 2.1	<10	0.007*	0.415
MUFA, E%	8.91 (6.99-11.7)	10–14	0.406	8.71 ± 3.33	10–14	0.058	0.366
PUFA, E%	5.4 ± 2.0	6–10	0.086	4.9 ± 1.5	6–10	<0.001**	0.263
Carbohydrate, E%	49.08 ± 7.42	45–60	0.012*	54.59 (47.4-56.39)	45–60	0.548	0.168
Total sugars, E%	11.8 ± 5.0	<10	0.044*	11.1 (8.7–16.5)	<10	0.079	0.710
Protein, g	82.53 (62.09-108.3)	–	NA	76.17 ± 23.74	–	NA	0.154
Animal protein, g	38.7 (32.4-49.6)	–	NA	37.31 ± 16.12	–	NA	0.343
Plant protein, g	42.98 (25.38-54.65)	–	NA	38.86 ± 18.66	–	NA	0.320
Fat, g	61.4 ± 19.6	–	NA	46.7 ± 17.3	–	NA	0.004*
SFA, g	23.4 ± 8.0	–	NA	18.2 ± 5.5	–	NA	0.004*
MUFA, g	24.1 ± 10.0	–	NA	16.0 (12.5–21.2)	–	NA	0.023*
PUFA, g	13.4 (8.5–17.4)	–	NA	10.3 ± 4.6	–	NA	0.018*
Cholesterol, mg	313.3 ± 121.7	<300	0.535	219.7 (144.4–315.5)	<300	0.005*	0.014*
Carbohydrate, g	297.35 ± 117.54	–	NA	259.21 (182.28-306.15)	-	NA	0.199
Total sugars, g	67.98 ± 33.12	–	NA	57.35 ± 25.71	-	NA	0.170
Dietary fiber, g	21.7 (16.0–32.5)	25–35	0.584	19.3 ± 7.9	25–35	0.001*	0.039*
Calcium, mg	818.47 (557.56-1337.82)	900	0.791	836.22 ± 382.38	900	0.403	0.444
Iron, mg	14.36 (12.65-26.56)	10	< 0.001	13.58 (9.46-21.6)	10	0.005	0.188
Copper, mg	1,79 ± 0,41	1,0	< 0,001	1,78 ± 0,6	1,0	<0,001	0,959
Zinc, mg	10.4 (8.6–13.2)	10	0.221	8.9 ± 2.9	10	0.062	0.022*
Vitamin A, µg RE	709.81 ± 311.93	900	0.001	522.76 (437.7-746.17)	700	0.099	0.221
Vitamin D, µg	3.6 (2.5–4.7)	10	<0.001**	2.5 (1.8–6.1)	10	<0.001**	0.255
Vitamin E, mg α-TE	12.98 ± 4.78	12	0.246	11.07 ± 4.71	10	0.258	0.129

Nutrient	Men (n=33)			Women (n=26)			P-value#
	Value	RDI	p-value	Value	RDI	P-value	
Vitamin B1, mg	1.46 (1.11–1.8)	1.2	0.004*	1.29 ± 0.49	1.0	0.006*	0.164
Vitamin B2, mg	1.81 (1.33–2.72)	1.4	<0.001**	1.5 (1.12–1.9)	1.2	<0.001**	0.108
Folic acid, µg	209.36 ± 52.19	200	0.311	202.52 (145.47–220.68)	200	0.822	0.49
Vitamin B6, mg	1.77 ± 0.58	1.6	0.105	1.62 ± 0.81	1.2	0.014*	0.439
Vitamin B12, µg	3.58 ± 1.7	3.0	0.061	3.68 ± 1.78	3.0	0.064	0.824
Vitamin C, mg	81.23 (55.32–103.32)	80	0.764	71.63 (54.81–85.65)	80	0.423	0.622

Values are given as mean ± standard deviation, median (interquartile range). The bold values indicate clinical significance. # p-value for the difference between men and women. MUFA, monounsaturated fatty acid; NA, not applicable; PUFA, polyunsaturated fatty acid; SFA, saturated fatty acid; RDI, recommended daily intake; * statistical significance at p value < 0.05, ** statistical significance at p value < 0.001.
